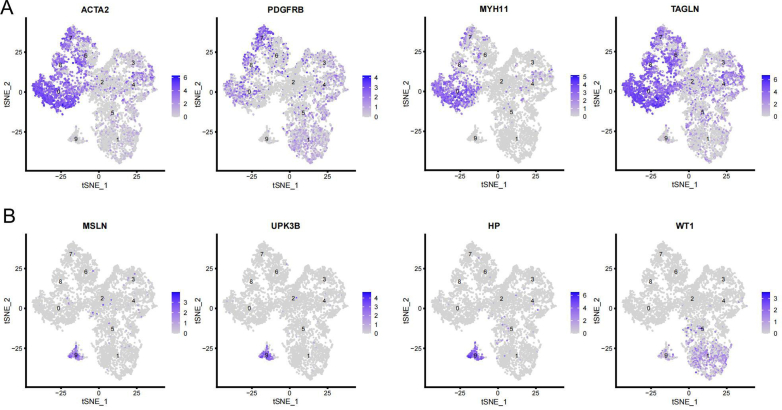# Prognostic model of fibroblasts in idiopathic pulmonary fibrosis by combined bulk and single-cell RNA-sequencing

**DOI:** 10.1016/j.heliyon.2024.e34519

**Published:** 2024-07-11

**Authors:** Jiarui Zhao, Chuanqing Jing, Rui Fan, Wei Zhang

**Affiliations:** College of First Clinical Medicine, Shandong University of Traditional Chinese Medicine, Jinan, Shandong, China

**Keywords:** Single-cell RNA-Sequencing, Bulk RNA-Sequencing, Fibroblast, Idiopathic pulmonary fibrosis, Prognostic model

## Abstract

**Background:**

Fibroblasts play an important role in the development of idiopathic pulmonary fibrosis (IPF).

**Methods:**

We employed single-cell RNA-sequencing data obtained from the Gene Expression Omnibus database to perform cell clustering and annotation analyses. We then performed secondary clustering of fibroblasts and conducted functional enrichment and cell trajectory analyses of the two newly defined fibroblast subtypes. Bulk RNA-sequencing data were used to perform consensus clustering and weighted gene co-expression network analysis. We constructed a fibroblast-related prognostic model using least absolute shrinkage, selection operator regression, and Cox regression analysis. The prognostic model was validated using a validation dataset. Immune infiltration and functional enrichment analyses were conducted for patients in the high- and low-risk IPF groups.

**Results:**

We characterized two fibroblast subtypes that are active in IPF (F3+ and ROBO2+). Using fibroblast-related genes, we identified five genes (*CXCL14*, *TM4SF1*, *CYTL1*, *SOD3*, and *MMP10*) for the prognostic model. The area under the curve values of our prognostic model were 0.852, 0.859, and 0.844 at one, two, and three years in the training set, and 0.837, 0.758, and 0.821 at one, two, and three years in the validation set, respectively.

**Conclusion:**

This study annotates and characterizes different subtypes of fibroblasts in IPF.

## 1 Introduction

Idiopathic pulmonary fibrosis (IPF) is a refractory form of interstitial lung disease. Generally, the incidence of IPF is higher in older males, with a history of cigarette smoking or occupational dust exposure [1,2]. IPF is a progressive disease, and untreated patients often experience a rapid decline leading to death [3]. Early IPF often presents with nonspecific clinical symptoms, such as a dry cough and dyspnea, whereas advanced IPF often causes respiratory and cardiac failure that can lead to death [4]. Therefore, distinguishing between different prognostic outcomes of IPF is an urgent clinical requirement. Fibroblast foci are an important pathological manifestation of IPF [5]. During abnormal processes, such as angiogenesis, apoptosis, and epithelial–mesenchymal transition, the extracellular matrix is extensively deposited in the lung interstitium [6]. These events ultimately lead to a decrease in lung compliance and exacerbation of IPF [7]. IPF is routinely treated with anti-fibrotic medications, such as nintedanib and pirfenidone [8]. Unfortunately, both drugs have a limited ability to control fibrosis progression and exhibit both tolerability and adverse effects [9,10]. Therefore, developing an IPF prognostic model that predicts disease progression and tailors treatment plans will be valuable in promoting precision medicine.

The exploration of IPF pathogenesis has led to increasing attempts to inhibit IPF progression by affecting fibroblast activity. Fibroblasts are components of the extracellular matrix that influence several physiological and pathological processes [11,12]. Fibroblasts can be classified into various types, including non-contractile fibroblasts and myofibroblasts [13]. Fibroblasts have the heterogeneity. Some fibroblast subtypes are closely related to the special tumor microenvironment and abnormal fibrotic changes [14]. A study has found that the THBS2+ fibroblasts subtype was significantly increased in lung adenocarcinoma, and the high expression of the THBS2+ fibroblasts subtype was closely associated with tumor growth and distant metastasis [15]. Fibroblasts in IPF are abnormally activated after stimulation by signals such as TGF-β [16]. Excessive deposition of the extracellular matrix leads to the pathological remodeling of IPF [17]. Fibroblasts play an important role in some processes such as oxidative reactions [18] and fiber foci migration [19] in IPF. Exploring the heterogeneity of fibroblasts in IPF is potentially beneficial in advancing the development of targeted therapies and cellular therapies in IPF. One study found that CD36 and CD96 were highly expressed in fibroblasts in the remodeling region of IPF [20]. There are limited studies related to fibroblasts in IPF. Considering the crucial role of fibroblasts in IPF, it is important to describe and explore the different subtypes of fibroblasts in IPF. The emergence of sequencing and microarray technologies has greatly advanced the annotation of different cell subtypes [21]. Moreover, the development of single-cell RNA-sequencing (scRNA-seq) technology allows us to study the status of each cell in disease from a more microscopic perspective [22] and fill the gaps in fibroblast annotation by screening for additional fibroblast-related prognostic genes.

We combined scRNA-seq with bulk RNA-sequencing (RNA-seq) analysis of IPF and control samples to explore different fibroblast subtypes and identify fibroblast-related prognostic genes. Using dimension reduction and subtype clustering from scRNA-seq data, we identified novel fibroblasts (F3+ and ROBO2+) that are active in IPF. We also characterized the cellular trajectories and biological functions of these newly defined fibroblast subtypes. Using fibroblast marker genes, we constructed a least absolute shrinkage and selection operator Cox (LASSO–COX) regression analysis using bulk RNA-seq data. Finally, we identified five IPF biomarkers and constructed a new fibroblast-related prognostic model. Our research advances the study of fibroblasts in IPF and provides an important reference for future basic research and clinical studies.

## 2 Results

### 2.1 Acquisition and annotation of Fibroblast subtypes

We retained only the scRNA-seq data from patients with IPF and the controls in GSE135893. After quality control and preprocessing (Supplementary Figs. 1A–B), we performed downscaling and clustering analyses and identified 29 cell subtypes (Fig. 1A). With reference to the marker genes from the original data [23], we categorized and annotated 29 cell subtypes and identified four common cell subtypes: epithelial cells, immune cells, fibroblasts, and endothelial cells (Fig. 1B). Marker genes for each of the four cell subtypes are shown in a bubble diagram (Fig. 1C), and the distribution of the four cell subtypes in different samples is shown in a bar chart (Supplementary Fig. 1D). We extracted individual fibroblasts and identified marker genes that distinguished them from other cell subtypes for subsequent analyses (Supplementary Table 1).

**Fig. 1:**
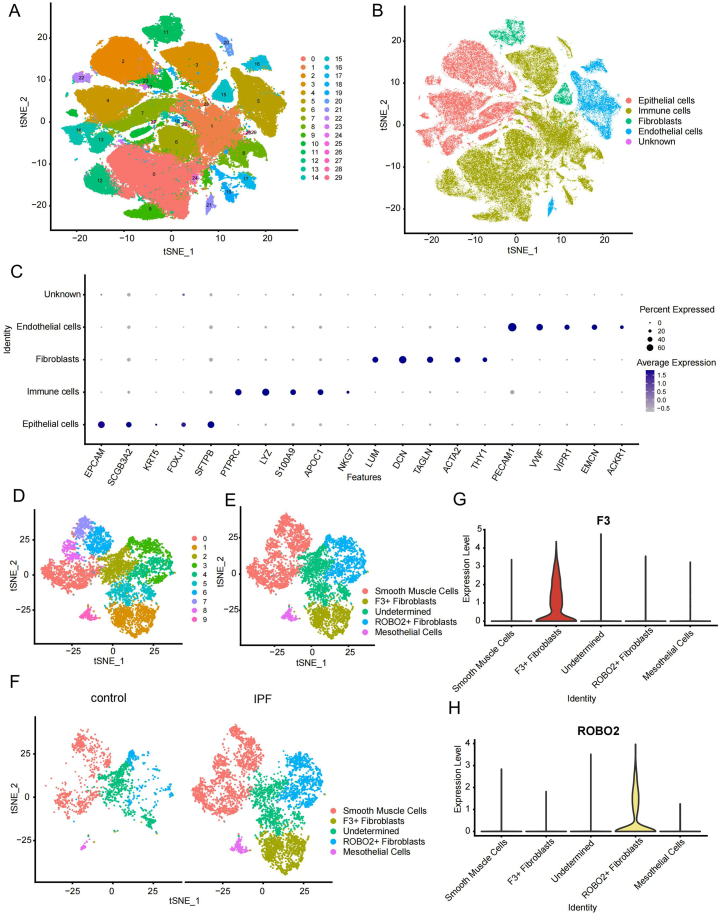
Clustering and annotation of single-cell subtypes. (**A**) Clustering subtypes of all cells were shown by t-SNE. (**B**) Annotation results of all cell subtypes, as shown by t-SNE. (**C**) Bubble plots of reference genes for annotation. Bubble size reflects the percentage of expression, and the more purple the bubble the higher the average expression. (**D**) Clustering subtypes of fibroblasts, as shown by t-SNE. (**E**) Annotation results of fibroblasts subtypes, as shown by t-SNE. (**F**) Annotation results of fibroblasts subtypes in different samples, as shown by t-SNE. (**G**) F3 expression in different fibroblast subtypes. (**H**) ROBO2 expression in different fibroblast subtypes. t-SNE, T-distributed stochastic neighbor embedding; F3+, F3 high expression; ROBO2+, ROBO2 high expression; IPF, idiopathic pulmonary fibrosis. (For interpretation of the references to color in this figure legend, the reader is referred to the Web version of this article.)

Following dimensional reduction and cluster analysis of the fibroblasts, we identified 10 fibroblast subtypes (Fig. 1D). Subtypes 0, 6, 7, and 8 were labeled as smooth muscle cells because they exhibited high expression of *ACTA2*, *PDGFRB*, *MYH11*, and *TAGLN*, which are smooth muscle cell marker genes (Supplementary Fig. 2A). Subtype 9 was labeled as mesothelial cells because it highly expressed the mesothelial cell marker genes *UPK3B*, *MSLN*, *HP*, and *WT1* (Supplementary Fig. 2B). Subtype 1 was typically found in IPF (Fig. 1F) and exhibited high expression of F3 Fibroblasts (Fig. 1G); therefore, subtype 1 was defined as F3+ Fibroblasts. Subtype 3 was typically found in IPF (Fig. 1F) and exhibited high *ROBO2* expression (Fig. 1H); therefore, subtype 3 was defined as ROBO2+ Fibroblasts. Finally, we obtained the annotation results for different fibroblast subtypes (Fig. 1E).

### 2.2 Description of F3+ fibroblasts and ROBO2+ fibroblasts

**Fig. 2:**
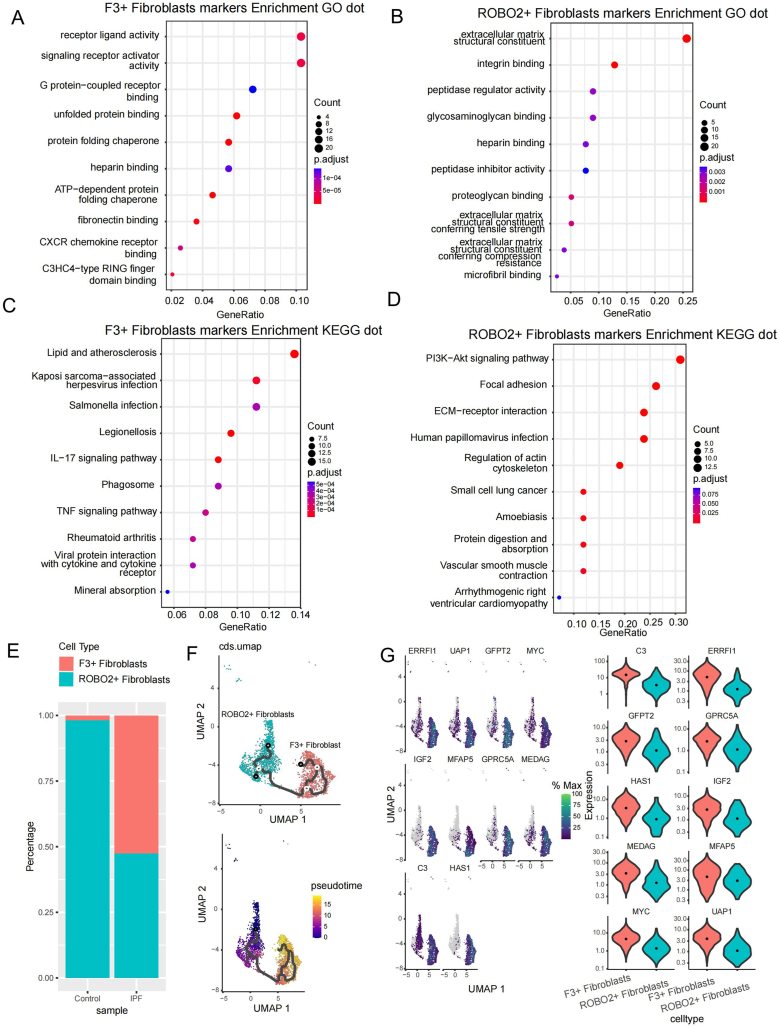
Description of defined fibroblast subtypes. GO analysis showing the biological processes in which (**A**) F3+ Fibroblasts and (**B**) ROBO2+ Fibroblasts were enriched. KEGG analysis showing the pathways in which (**C**) F3+ Fibroblasts and (**D**) ROBO2+ Fibroblasts were enriched. (**E**) Percentage of F3+ Fibroblasts and ROBO2+ Fibroblasts in different samples. The larger the bubble the higher the correlation, and the redder the bubble the smaller the P-value. (**F**) Cell trajectory analysis from ROBO2+ Fibroblasts to F3+ Fibroblasts. (**G**) Top 10 genes with the most significant changes from ROBO2+ Fibroblasts to F3+ Fibroblasts. GO, Gene Ontology; F3+, F3 high expression; KEGG, Kyoto Encyclopedia of Genes and Genomes; ROBO2+, ROBO2 high expression; IPF, idiopathic pulmonary fibrosis; UMAP, uniform manifold approximation and projection.

We further explored and characterized the newly defined F3+ and ROBO2+ fibroblasts. Gene Ontology (GO) analysis suggested that F3+ Fibroblasts were associated with receptor ligand activity, G protein-coupled receptor binding, signaling receptor activator activity, and unfolded protein binding (Fig. 2A), whereas ROBO2+ fibroblasts were associated with extracellular matrix structural constituents, integrin binding, peptidase regulator activity, and glycosaminoglycan binding (Fig. 2B). Kyoto Encyclopedia of Genes and Genomes (KEGG) enrichment analysis suggested that F3+ Fibroblasts were associated with the lL-17 and TNF signaling pathways (Fig. 2C), whereas ROBO2+ fibroblasts were associated with the PI3K-AKt and focal adhesion signaling pathways (Fig. 2D).

Compared with ROBO2+ Fibroblasts, F3+ Fibroblasts were more commonly present in IPF (Fig. 2E). Using ROBO2+ Fibroblasts as the initial stage of our trajectory, we performed cell trajectory analysis of the two fibroblast subtypes (Fig. 2F). We calculated the differentially expressed genes (DEGs) from F3+ Fibroblasts to ROBO2+ Fibroblasts (Supplementary Table 2) and showed the top 10 genes (*C3*, *ERRFI1*, *UAP1*, *GFPT2*, *MYC*, *IGF2*, *MFAP5*, *GPRC5A*, *MEDAG*, *HAS1*) with the most significant changes over pseudotime (Fig. 2G).

### 2.3 Consensus clustering analysis based on Fibroblast marker genes

**Fig. 3:**
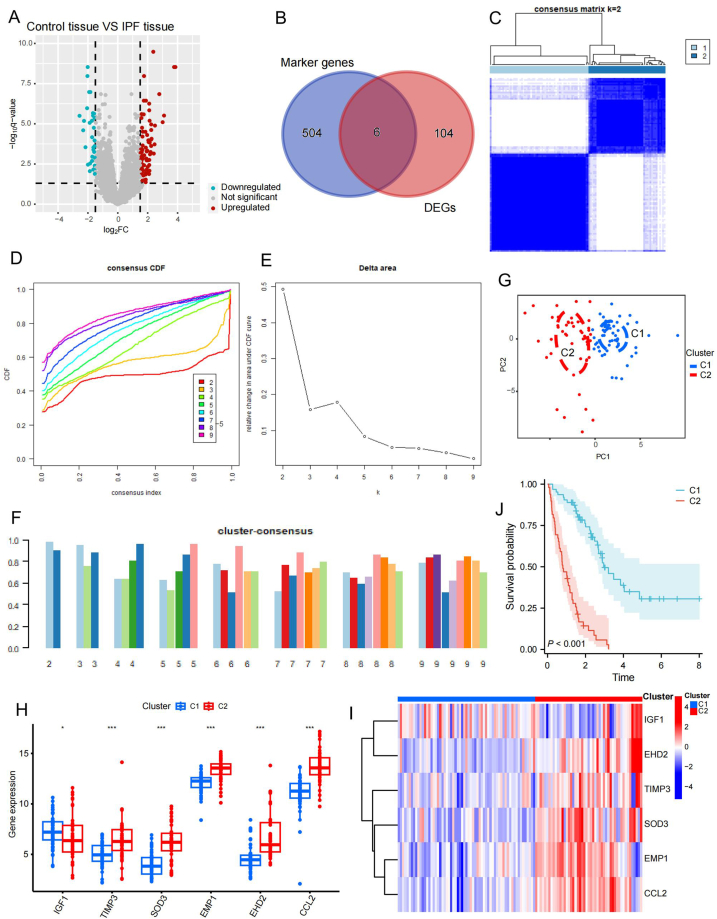
Consensus clustering analysis based on fibroblast marker genes. (**A**) Volcano plot showing the 110 DEGs of IPF and control samples in the training set. DEGs were filtered using log2FC > 1.5 and a Benjamini–Hochberg-adjusted P value of <0.05. The red dots reflect genes that were upregulated in the IPF, and the blue dots represent genes that were downregulated in the IPF. (**B**) Intersection of 510 fibroblast marker genes with 110 DEGs revealed six differentially expressed fibroblast marker genes. (**C**) Distribution of the consensus matrix for K = 2. (**D**) Different degrees of decline for CDF curves for different values of K when the consensus index changed from 0.9 to 0.1. Colors of the curves represent different K values from 2 to 9. (**E**) Delta area plot showed variations in the area under the CDF curve. (**F**) Bar diagram showing the different conditions of K from 2 to 9. (**G**) PCA plot demonstrating the ability of C1 and C2 to separate IPF samples. **(H)** Different expression of six fibroblast marker genes between C1 and C2. Blue for C1 and red for C2. (**I**) Heatmap showing the specific expression condition of the six fibroblast marker genes in C1 and C2. **(J)** Survival curves showing prognostic differences between C1 and C2 (P < 0.01). log2FC, absolute value of fold change; CDF, cumulative distribution function; PCA, principal component analysis; IPF, idiopathic pulmonary fibrosis; C1, Cluster 1; C2, Cluster 2; DEGs, differentially expressed genes. *P < 0.05; ***P < 0.001. (For interpretation of the references to color in this figure legend, the reader is referred to the Web version of this article.)

The Freiburg, Germany, and Siena, Italy cohorts from the GPL14550 probe platform were used for the difference analysis. A total of 110 DEGs (Fig. 3A) were obtained from the differential analysis (Supplementary Table 3). These 110 DEGs was intersected with 510 fibroblast marker genes. In order to obtain a prognostic model with good clinical value, we used marker genes from all fibroblasts for subsequent analysis. Then we got six differentially expressed fibroblast marker genes: *IGF1*, *TIMP3*, *EHD2*, *SOD3*, *EMP1*, and *CCL2* (Fig. 3B). These six fibroblast marker genes were used for subsequent consensus cluster analysis. We tested different values of K ranging from 2 to 9 to determine the optimal value of K. The consensus matrix plot indicated a good clustering effect for K = 2 (Fig. 3C). Considering the change in the cumulative distribution function (CDF) curve when the consensus index decreased from 0.9 to 0.1, we also considered two as the optimal K value (Fig. 3D and E). Moreover, the bar diagram shows the best grouping for K = 2 (Fig. 3F). Through consensus clustering analysis, we obtained clusters 1 (C1) and 2 (C2). The principal component analysis (PCA) plot suggested that the clustering effect was good and that C1 and C2 could effectively distinguish the IPF samples (Fig. 3G). According to the bar diagram, the six fibroblast marker genes used for clustering showed significant differences in expression between C1 and C2 (Fig. 3H). The volcano plot demonstrates the specific expression conditions of the six fibroblast marker genes in C1 and C2 (Fig. 3I). To explore whether there are differences in the clinical characteristics of C1 and C2. We performed a prognostic analysis of C1 and C2 using clinical survival information. We found a significant difference in survival outcome between C1 and C2. The prognostic outcome of C2 was significantly worse than that with C1 (P < 0.01, Fig. 3J). This outcome shows that our clustering is good, and that there is a link between fibroblast activities and patient prognosis.

### 2.4 WGCNA and LASSO–COX analysis

**Fig. 4:**
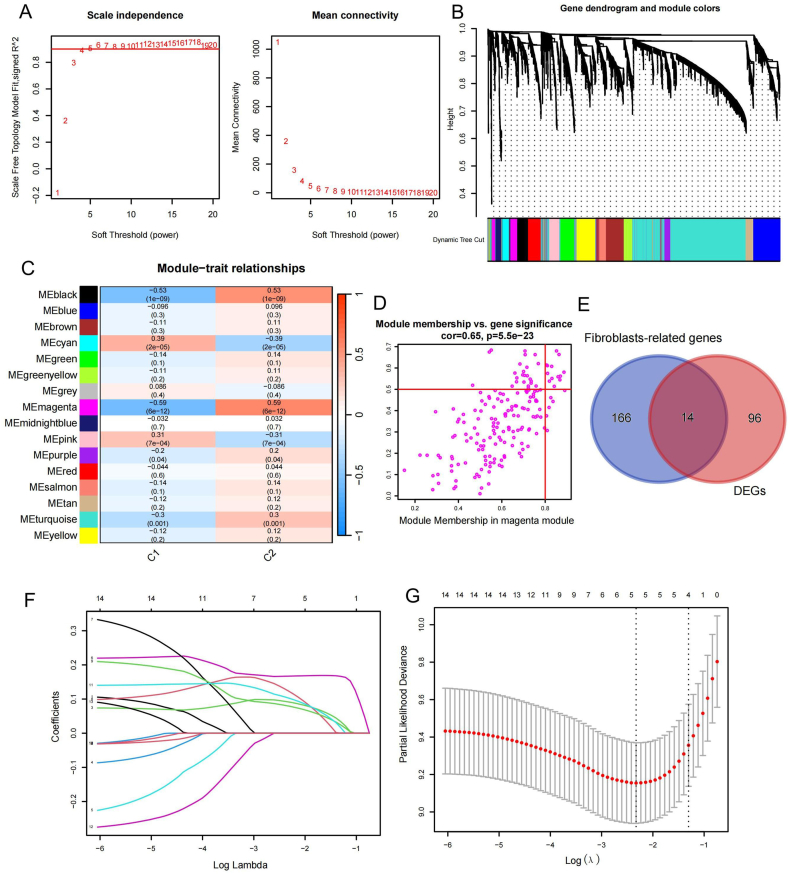
WGCNA analysis was conducted to obtain fibroblast-related gene modules and LASSO–COX analysis was conducted to obtain prognostic model genes. (**A**) With different soft thresholds, we obtained different scale-free fit index and network connectivity. The best soft threshold (4) was obtained when the scale-free fit index was >0.9. (**B**) Tree diagram showing the different gene modules obtained from clustering. (**C**) Module–trait relationship heatmap showing P-values and correlations of different color modules. The magenta module had a small P value (P = 6e-12) and a high correlation. (**D**) Scatter plot of the correlation between module membership and gene significance in the magenta module (correlation = 0.65, P = 5.5e-23). (**E**) Intersection of 180 genes in the key magenta module with 110 DEGs revealed 14 DEGs associated with fibroblasts. (**F**) Coefficient distribution of 14 fibroblast-related genes under LASSO–COX regression analysis. (**G**) Regression parameters of LASSO–COX. Optimal genes were obtained when the parameter was set to lambda.min. C1, Cluster 1; C2, Cluster 2; DEGs, differentially expressed genes. (For interpretation of the references to color in this figure legend, the reader is referred to the Web version of this article.)

To identify additional genes related to fibroblasts, we performed weighted gene co-expression network analysis (WGCNA) on C1 and C2. Genes with the top 15 % of variance were used for co-expression network module construction. The combined scale independence and mean connectivity plots for the analysis showed that the optimal soft threshold was four (Fig. 4A); therefore, this threshold was used to present the differently clustered modules using a clustering tree (Fig. 4B). According to the heatmap of module–trait relationships, the magenta module had a small P-value (P = 6e-12) and a high correlation (Fig. 4C). The correlation between gene significance and membership in the magenta module was high (correlation = 0.65, P = 5.5e-23; Fig. 4D). Finally, we identified the magenta module as the key module. The 180 genes in the magenta module (Supplementary Table 4) were intersected with 110 DEGs to obtain 14 fibroblast-related genes (Supplementary Table 5; Fig. 4E). Based on these 14 fibroblast-related DEGs, we performed LASSO–COX regression analysis (Fig. 4F). The best-characterized genes were obtained when lambda was five (Fig. 4G). Based on the best-characterized genes (*CXCL14*, *TM4SF1*, *CYTL1*, *SOD3*, and *MMP10*) and their correlation coefficients, we developed a prognostic model using the following risk-score formula:

RiskScore=CXCL14×(0.091615149789772)+TM4SF11×(0.167011408782904)+CYTL1×(0.123800961826157)+SOD3×(0.0796322777533957)+MMP10×(0.103184037393196)

### 2.5 Construction of the fibroblast-related prognostic model

**Fig. 5:**
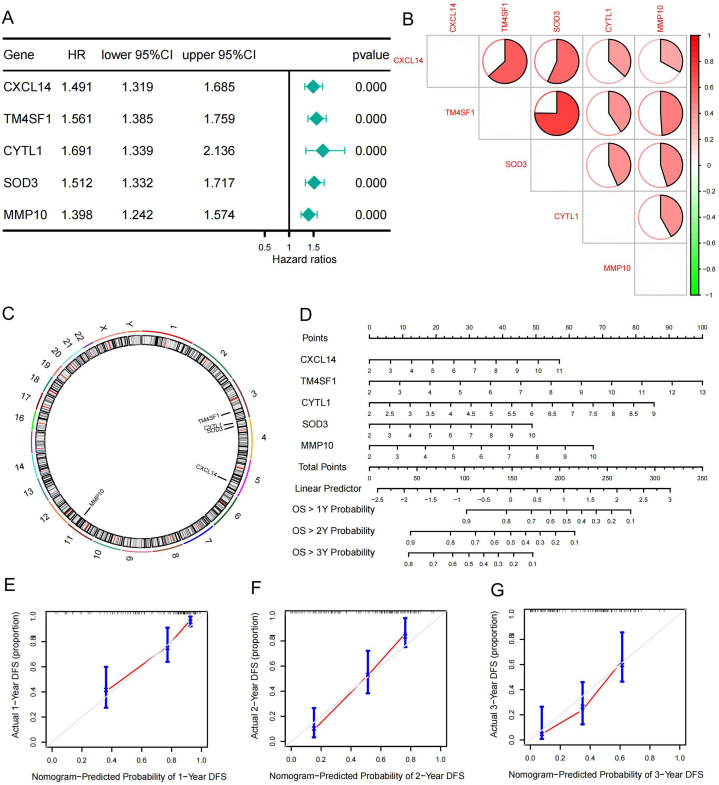
Construction of the fibroblast-related prognostic model. (**A**) Univariate Cox analysis for the genes in each model revealed that gene P-values were all less than 0.000. (**B**) Correlation analysis between model genes indicated positive correlations among all genes. The redder the color the higher the positive correlation. (**C**) Chromosome circle demonstrating the location of the model genes. (**D**) Nomogram plot showing the condition of the model at one, two, and three years. Calibration curves of the prognostic model at (**E)** one year, (**F**) two years, and (**G**) three years. CI, confidence interval; HR, hazard ratio. (For interpretation of the references to color in this figure legend, the reader is referred to the Web version of this article.)

The results of univariate Cox suggested that *CXCL14* (hazard ratio [HR] = 1.491, 95 % confidence interval [CI]: [(1.319, 1.685], P < 0.001), *TM4SF1* (HR = 1.561, 95%CI: [(1.385, 1.759], P < 0.001), *CYTL1* (HR = 1.691, 95%CI: [(1.339, 2.136], P < 0.001), *SOD3* (HR = 1.512, 95%CI: [(1.332, 1.717], P < 0.001) and *MMP10* (HR = 1.398, 95%CI: [(1.242, 1.574], P < 0.001) all had independent prognostic ability (Fig. 5A). Correlation analysis was performed to investigate the correlations between the five model genes. All five genes were positively correlated, and the strongest correlation was found between *TM4SF1* and *SOD3* (correlation = 0.75; Fig. 5B). We then drew a chromosome circle to show the locations of the five model genes on the chromosomes (Fig. 5C). We found that *TM4SF1* is located on chromosome 3, *CYTL1* and *SOD3* on chromosome 4, *CXCL14* on chromosome 5, and *MMP10* on chromosome 11. The fibroblast-related prognostic model was then constructed using the five model genes. A nomogram (Fig. 5D) and calibration curve plots (Fig. 5E–G) were used to demonstrate the predictive ability of our prognostic model for one to three years.

### 2.6 Internal evaluation and external validation

**Fig. 6:**
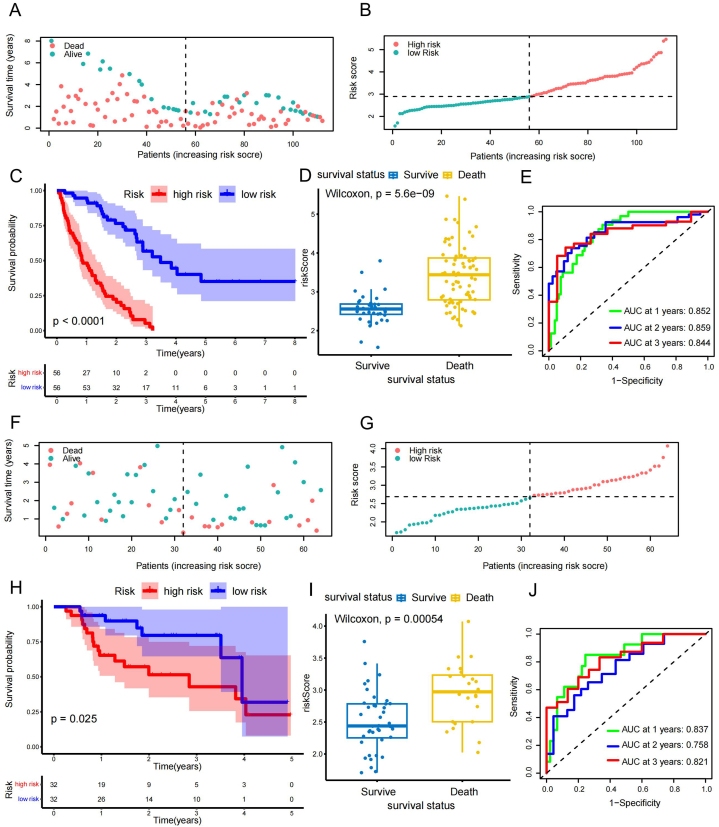
Internal evaluation and external validation. (**A**) Dot plot showed the survival status of all samples in the training set. Survival in green and death in red. (**B**) Median value of risk score in the training set was used to distinguish high-risk-score patients from low-risk-score patients. (**C**) Survival curves showing prognostic differences between patients with different risk scores in the training set (P < 0.000). (**D**) Wilcoxon test revealed different prognostic endings between patients with different risk scores in the training set (P = 5.6e-9). (**E**) TimeROC curves for the training set, reflecting the good prognostic abilities of the model at one year (AUC = 0.852, 95%CI: [0.783, 0.922]), two years (AUC = 0.859, 95%CI: [0.785, 0.934]), and three years (AUC = 0.844, 95%CI: [0.753, 0.936]). (**F**) Plot of the survival status of all samples in the validation set; survival is shown in green and death is shown in red. (**G**) Median risk score in the validation set was used to distinguish high-risk-score patients from low-risk-score patients. (**H**) Survival curves showing prognostic differences between patients with different risk scores in the validation set (P = 0.025). (**I**) Wilcoxon test revealed different prognostic endings between patients with different risk scores in the validation set (P < 0.001). (**J**) TimeROC curves for the validation set reflecting the good prognostic abilities of the model at one year (AUC = 0.837, 95%CI: [0.721, 0.953]), two years (AUC = 0.758, 95%CI: [0.609, 0.908]), and three years (AUC = 0.821, 95%CI: [0.679, 0.962]). AUC, area under the curve; CI, confidence interval; HR, hazard ratio; ROC, receiver operating characteristic. (For interpretation of the references to color in this figure legend, the reader is referred to the Web version of this article.)

The risk scores of different survival status samples in the training set are presented as dot plots (Fig. 6A). The median risk score in the training set was used as a boundary to divide high-risk and low-risk patients (Fig. 6B). The survival curves illustrated that the prognosis of patients in the high-risk score group in the training set was significantly worse than that of patients in the low-risk score group (P < 0.001; Fig. 6C). The box plot demonstrated a significant difference between patients with different prognostic statuses (P = 5.6e-09; Fig. 6D). The time-dependent receiver operating characteristic (timeROC) curves for the training set indicated good prognostic abilities of our model at one year (Area under the curve [AUC] = 0.852, 95%CI: [0.783, 0.922]), two years (AUC = 0.859, 95%CI: [0.785, 0.934]), and three years (AUC = 0.844, 95%CI: [0.753, 0.936]) (Fig. 6E). Multifactorial Cox analysis of the risk score in the training set, combined with two common clinical factors (age and sex; Supplementary Table 6), showed that our risk score had independent prognostic power (Table 1).

**Table 1:** Multivariate Cox analysis.

Multivariate Cox analysis	Age		Gender		Risk score	
	Hazard ratio	P−value	Hazard ratio	P−value	Hazard ratio	P−value
Training set	0.990	0.296	1.490	0.233	1.420	<0.001
Validation set	1.000	0.147	1.200	0.784	3.900	0.003

The survival status and risk scores of the different samples in the validation set are presented as dot plots (Fig. 6F). The median risk score in the validation set was used as a boundary to distinguish between patients with high- and low-risk scores (Fig. 6G). As with the training set, the survival curves illustrate that the prognosis of patients in the high-risk score group in the validation set was significantly worse than that of patients in the low-risk score group (P = 0.025; Fig. 6H). The box plot also demonstrated a significant difference between patients with different prognostic statuses (P < 0.001) in the validation set (Fig. 6I). The timeROC for the validation set indicated good prognostic abilities of our model at one year (AUC = 0.837, 95%CI: [0.721, 0.953]), two years (AUC = 0.758, 95%CI: [0.609, 0.908]), and three years (AUC = 0.821, 95%CI: [0.679, 0.962]) (Fig. 6J). We again performed a multifactorial Cox analysis of the risk score in the validation set, combined with two common clinical factors (age and sex; Supplementary Table 7), and again found that the risk score had independent prognostic power (Table 1).

### 2.7 Description of high- and low-risk-score groups

**Fig. 7:**
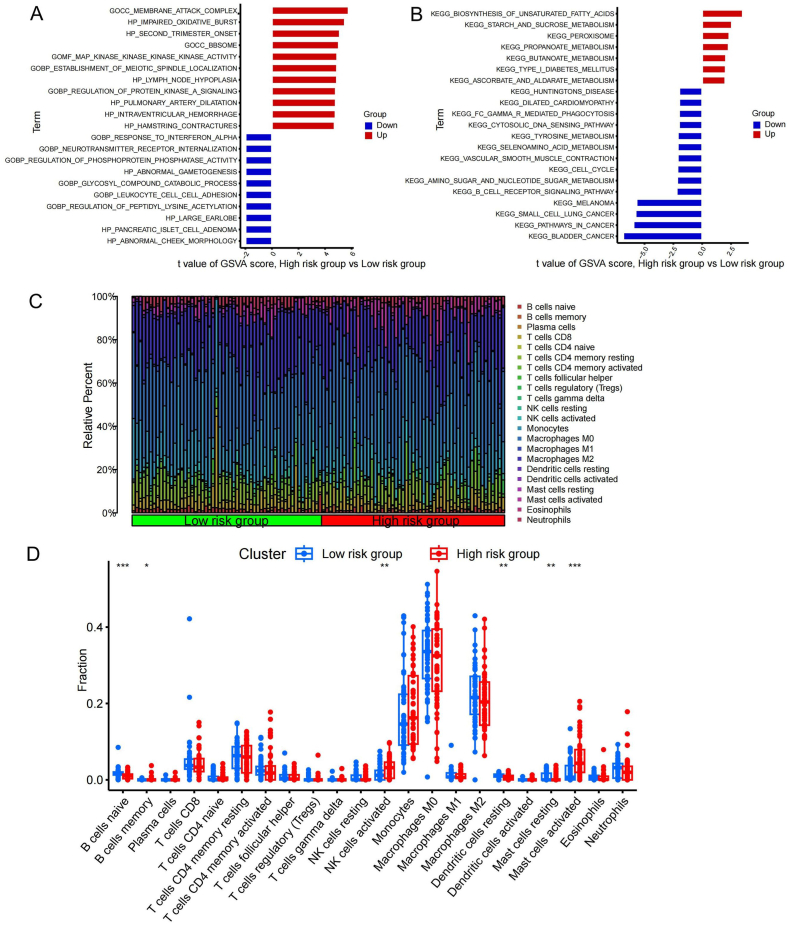
Description of the respective immune infiltrates and biological functions of different risk score groups. (**A**) GO enrichment analysis and (**B**) KEGG enrichment analysis between patients with different risk scores. (**C**) Abundance of immune infiltrations in nts with different risk scores. (**D**) Expression differences of 22 immune cells between nts with different risk scores. KEGG, Kyoto Encyclopedia of Genes and Genomes; GO, Gene Ontology; GSVA, gene set variation analysis. *P < 0.05; **P < 0.01; ***P < 0.001.

Based on the fibroblast-related prognostic model, IPF was classified into different risk-scoring groups, and gene set variation analysis (GSVA) was performed for both groups. Processes such as impaired oxidative burst and membrane attack complexes, and signaling pathways such as the biosynthesis of unsaturated fatty acids and starch and sucrose metabolism, were more active in high-risk patients (Fig. 7A and B). Patients with high-risk scores were more metabolically active than those with low-risk scores. This finding suggests that fibroblast activity and poor IPF prognosis may be related to metabolism.

Using CIBERSORT analysis, we determined the relative abundance of 22 immune cell infiltrates in IPF (Fig. 7C). We found that the activities of natural killer and mast cells were significantly higher in patients with high-risk scores , whereas the activities of naive and memory B cells were significantly lower (Fig. 7D). Therefore, poor IPF prognosis may be closely related to changes in the activities of immune cells, such as B cells, natural killer cells, and mast cells.

## 3 Discussion

IPF occurs in approximately 50 out of every 100,000 individuals, and its incidence is positively correlated with age [17,24]. Continued damage and scarring of the lung tissue in IPF may be associated with many factors, such as the environment and genes [25,26]. Acute exacerbation of IPF leads to the rapid deterioration of lung function and accelerates patient death [27]. Unfortunately, identifying the causative factors in most cases of IPF deterioration is challenging [28]. Therefore, identifying additional methods for predicting IPF prognosis in clinical practice is urgently required. Fibroblasts play an important role in the development of IPF; a large number of fibrotic foci are present in IPF and fibroblasts are crucial for maintaining cellular structure [29]. In IPF, cytokine stimulation largely activates fibroblasts [30]. The extracellular matrix is modified and deposited, leading to the progression of fibrotic foci in IPF. Fibroblasts exhibit diverse subtypes in different states [31]; however, there are gaps in the annotation of different fibroblast subtypes in IPF. Therefore, by studying the function and expression of different fibroblast subtypes in IPF, our study represents an advance in IPF research. Recently, an increasing number of IPF biomarkers have been identified, with studies revealing *S100A12*, *MMP7*, and *CXCL13* as potential prognostic markers for IPF [[32], [33], [34]]. Unfortunately, these markers do not meet the clinical requirements for IPF diagnosis or treatment. Therefore, additional IPF biomarkers with good prognostic ability must be identified. In this study, we used scRNA-seq technology, which provides an opportunity to explore fibroblasts in IPF, to construct a fibroblast-related prognostic model for IPF.

By clustering and annotating scRNA-seq data from the Gene Expression Omnibus (GEO) database, we obtained fibroblast subtypes. We performed further detailed clustering of the obtained fibroblasts and identified ROBO2+ and F3+ fibroblasts as active fibroblasts in IPF. Due to high expression of ROBO2+ gene, ROBO2+ fibroblasts are labeled with ROBO2+. F3+ fibroblasts are labeled with F3 due to high expression of the F3 gene. ROBO2 encodes a protein that is a part of the ROBO family, which is involved in SLIT-ROBO signaling and plays a role in cell migration and tumor metastasis [35]. F3, also known as CD142, is closely associated with inflammation, mesenchymal reorganization and blood coagulation [36]. One study had defined a tumor-associated fibroblast subpopulation with high F3 expression in colon cancer, which can secrete more HGF factors and promote tumor proliferation by activating various pathways such as RAS or PI3K [37]. To explore the functional characteristics of these two fibroblast subtypes in IPF, we performed GO and KEGG analyses on both ROBO2+ Fibroblasts and F3+ Fibroblasts. The results showed that ROBO2+ Fibroblasts were closely related to extracellular matrix construction and the PI3K-AKT signaling pathway. It has been found that ROBO2 membrane receptor can promote the progression of liver fibrosis by activating the PI3K-AKT pathway upon binding to the ligand SLIT2 [38]. The PI3K-AKT pathway is closely related to IPF progression, and it can influence some processes such as epithelial-mesenchymal transition (EMT) and apoptosis to ultimately fibrosis progression [39]. So the activation of the PI3K-AKT pathway may be a potential mechanism by which ROBO2+ fibroblasts affect extracellular matrix reconstitution in IPF. The F3+ Fibroblasts were closely associated with the IL-17 signaling pathway, inflammatory response, and the TNF signaling pathway. A previous study found that TNF signaling can affect fibroblast activity in IPF [40], and another found that IL-17 family members affect IPF progression [41]. We believe that the progression of IPF is closely related to inflammatory activity in fibroblasts. Modulating the inflammatory response associated with F3+ Fibroblasts could potentially be beneficial in controlling the development of IPF. Moreover, we combined ROBO2+ and F3+ fibroblasts for cell trajectory analyses. Because F3+ Fibroblasts were more active in IPF, we set this as the endpoint of our cell trajectory. We observed some heterogeneity between ROBO2+ and F3+ fibroblasts [42]. Furthermore, we demonstrated that 10 genes (*C3*, *ERRFI1*, *UAP1*, *GFPT2*, *MYC*, *IGF2*, *MFAP5*, *GPRC5A*, *MEDAG*, and *HAS1*) showed the most pronounced changes over the pseudotime of our cell trajectory, and that F3+ Fibroblasts were associated with inflammatory responses.

Using fibroblast marker genes, we performed consensus clustering analysis on the bulk RNA-seq data. After performing WGCNA on the consensus clustering results, we identified 180 genes with the highest correlation with fibroblasts in IPF. By examining the intersection of 180 genes with 110 DEGs, we identified 14 fibroblast-related DEGs that were subsequently used for LASSO–COX analysis, revealing five model genes: *CXCL14*, *TM4SF1*, *CYTL1*, *SOD3*, and *MMP10*. After verifying the prognostic ability of these five model genes, we explored their correlation and determined their distribution on the chromosomes. We found a strong positive relationship between *TM4SF1* and *SOD3* and attempted to analyze the potential mechanism of IPF. Specifically, we constructed a fibroblast-related prognostic model and risk score formula for these five model genes, then plotted nomograms and calibration curves for the proposed model. Based on the median risk score, we categorized the training and validation sets into different risk-score groups. We then evaluated our model for the training and validation sets using survival, receiver operating characteristic (ROC), and multivariate Cox analyses. One study found 14 prognostic features based on fibroblasts in IPF [43]. However, our prognostic model only contained 5 model genes. The number of genes in our model is more practical for clinical application and can be better applied for clinical evaluation. Through time-dependent ROC curve analysis, we evaluated and calculated the AUC values of our prognostic model at year 1, year 2 and year 3. Compared with previous studies, the AUC values of our model at 1 and 3 years were higher than 0.800 in both validation and training set data. Our prognostic model has excellent evaluation ability to predict and assess the trend of disease change and guide personalized treatment. Currently, there are fewer high-quality prognostic model in IPF, so our model has some important clinical value.

Based on the obtained prognostic model, we distinguish patients with different prognostic risks. There were significant differences in the functional enrichment analysis and immune infiltration analysis in high-risk patients compared to low-risk patients. The functional enrichment analysis revealed that abnormal oxidative and metabolic responses were activated in the high-risk score group. This recommends that we ameliorate the occurrence of abnormal oxidative stress in IPF is in favor of controlling IPF progression. The immune infiltration analysis revealed that mast and natural killer cells were active in the high-risk score group, whereas naive and memory B cells were active in the low-risk score group. The activities of mast cells in IPF have been described previously [44]. Therefore, there is an opportunity to control the progression of IPF by affecting the expression of immune cells such as mast cells, B cells, and natural killer cells in IPF treatment. Our exploration provides some help in predicting patient prognosis and guiding individualized treatment for different patients.

The five model genes used were *CXCL14*, *TM4SF1*, *CYTL1*, *SOD3*, and *MMP10*. CXCL14 is a member of the cytokine family, which influences immune cell activity. CXCL14 may affect fibroblast activation in IPF through the CXCL12/CXCL14 axis [45], as well as IPF progression through the Hedgehog signaling pathway [46]. *TM4SF1* encodes a transmembrane protein, TM4SF1, that participates in intercellular signaling. TM4SF1 can also participate in EMT through the WNT signaling pathway [47] and fibroblast activity through the AKT/ERT signaling pathway [48]. *CYTL1* encodes a cytokine-like 1 protein that is closely related to inflammatory regulation. The expression of CYTL1 is closely associated with cardiac fibrosis [49]. *SOD3* encodes superoxide dismutase 3, which regulates oxidative homeostasis and can influence the progression of IPF by decreasing oxidative load [50]. Finally, *MMP10*, a gene encoding Matrix Metallopeptidase 10, is involved in the degradation and remodeling of the extracellular matrix. A link has been observed between the expression of MMP10 and IPF [51].

Our manuscript has a certain value and novelty. We first identified ROBO2+ and F3+ fibroblasts and advanced their functional characterization in IPF. In addition, we developed a fibroblast-related prognostic model and explored novel IPF biomarkers. The AUCs of our model, both for the training and validation sets, exceeded 0.8 at both one and three years. Our findings are valuable for future research on IPF diagnosis and treatment. Our manuscript also has some limitations. Our study focuses on exploring a high-quality prognostic model, so the exploration of the molecular mechanisms of the new fibroblast subtypes were limited. Our study has some reliance on publicly available datasets and also potentially has some technically unavoidable biases. Future research will involve specific experiments to evaluate the mechanism of fibroblast activity in IPF.

## 4 Materials and methods

### 4.1 Quality control and preprocessing of raw data

Raw scRNA-seq data were obtained from the GSE135893 dataset in the GEO database (https://www.ncbi.nlm.nih.gov/gds) [23]. The GSE135893 dataset contained 20 diseased and 10 control lung samples. Twelve IPF and 10 control scRNA-seq data points were retained for subsequent analyses. scRNA-seq data were filtered using the R "Seurat" package [52] with the criterion of "min. features less than 200." We then normalized scRNA-seq data using the "NormalizeData" function. Using the "ScaleData" function, we reduced the bias of scRNA-seq data caused by mitochondrial genes and cell cycles and selected the highest 2000 mutated genes using the "vst" method to identify different cell subtypes.

**Fig. 8:**
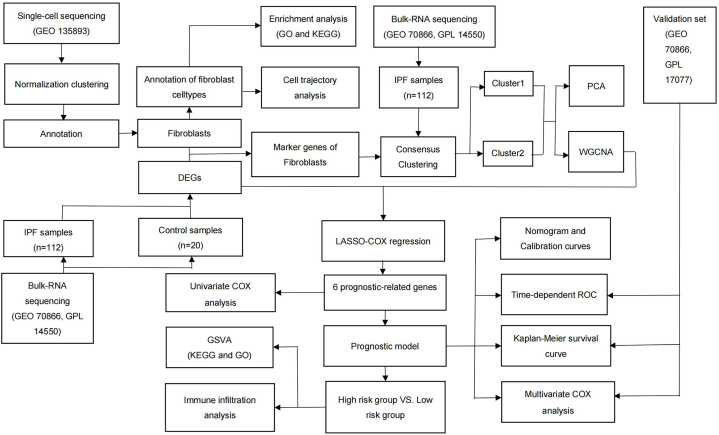
Flow diagram showing the study procedure.

Raw bulk RNA-seq data were obtained using the "GEOquery" R package [53] from the GSE70866 dataset in the GEO database [54]. The GSE70866 dataset comprised three separate cohorts from three different regions. The Freiburg, Germany cohort (62 IPFs and 20 controls) was obtained from the same probe platform (GPL14550) as the Siena, Italy cohort, both of which were used as the training set. The Leuven, Belgium (64 patients) dataset was obtained from another probe platform (GPL17077) and used as the validation set. The "limma" R package [55] was used for standardized preprocessing of the raw bulk RNA-seq data (Supplementary Fig. 1F). We used the organized data for subsequent analyses. The study procedure is illustrated in Fig. 8.

### 4.2 Clustering and annotation

Dimensional reduction of single-cell data was performed using the R "Seurat" package [52]. The "harmony" package [56] was used to deal with the batch effects. In order to reduce the technical noise, we use elbow point to select the appropriate value of principal components (PCs) for clustering. Because the PCs were relatively smooth when reduced to 50, a value of 50 was chosen for cell clustering (Supplementary Fig. 1C). The "FindNeighbors" and "FindCluster" functions were used for cell clustering. The parameter resolution was set to 0.5 to obtain the cell clustering results using the T-distributed stochastic neighbor embedding (t-SNE) method. According to relevant literature [23], we annotated the cell subtypes and obtained five subtypes by referencing the relevant literature. According to the following settings: "only.pos = T," "logfc. threshold = 0.25," and "min. pct = 0.25," we obtained marker genes for each of the five identified cell subtypes, including fibroblasts.

The fibroblasts were selected for further downscaling and clustering. The elbow point was used to select appropriate PCs for further clustering of fibroblasts. The value of 20 was chosn for cell clustering, because the PCs were relatively smooth when reached to 20 (Supplementary Fig. 1E). To obtain clustering results for fibroblasts, the parameter resolution was set to 0.5. The results are shown using t-SNE. The following settings were used to obtain the highly expressed genes for different fibroblast subtypes according to the "FindAllMarkers" function: "only.pos = T," "logfc.threshold = 0.25," and "min.pct = 0.25." Different fibroblasts were annotated based on their highly expressed genes.

### 4.3 Functional enrichment analysis and cell trajectory analysis

We first identified an F3+ Fibroblasts and a ROBO2+ Fibroblasts. Both fibroblast subtypes were active in the IPF samples. We then obtained marker genes of F3+ Fibroblasts using a Benjamini–Hochberg-adjusted P-value of less than 0.01 and a fold change value (log2FC) of greater than one. We also obtained marker genes of ROBO2+ Fibroblasts with a Benjamini–Hochberg-adjusted P-value of less than 0.01 and a log2FC value of less than one. GO and KEGG enrichment analyses were performed for F3+ Fibroblasts and ROBO2+ Fibroblasts using the R "clusterProfiler" package [57]. To explore the relationship between F3+ Fibroblasts and ROBO2+ Fibroblasts, we conducted cell trajectory analysis using the R "monocle3″ package [[58], [59], [60]]. The cell trajectory was constructed using the "learn_grap" function. Since ROBO2+ Fibroblasts were less distributed in the IPF, ROBO2+ Fibroblasts were set as the starting point of the trajectory.

### 4.4 Consensus clustering analysis

The training set RNA-seq data was analyzed by the "limma" R package [55]. DEGs were screened when the absolute log2FC was greater than 1.5 and the Benjamini–Hochberg-adjusted P-value was less than 0.05. By intersecting the 110 DEGs with fibroblast marker genes, we identified six DEGs. The six fibroblast marker genes were then used to perform consensus clustering analysis of the IPF samples using the R "ConsensusClusterPlus" package [61] and k-means clustering. Set the "ConsensusClusterPlus" function: "maxK = 9″, "PItem = 0.8″, "pFeature = 1″, "clusterAlg = km" and "distance = euclidean". According to the combined consensus CDF plot, delta area plot, consensus matrix heatmap, and cluster-consensus plot, the optimal K value from one to nine was two. The effectiveness of the consensus clustering was evaluated using a PCA plot.

### 4.5 Weighted gene Co-expression network analysis

Following consensus clustering analysis, the IPF samples of the training set were divided into clusters 1 and 2. The top 15 % of mutated genes in the RNA-seq data were subjected to WGCNA using the "WGCNA" package [62]. The most outstanding soft thresholding power value was determined through the "pickSoftThreshold" function. The obtained soft-threshold value was then used for network construction. Clustered modules were identified using the topological overlap measure, and different co-expressed gene modules were randomly assigned different colors. Finally, we selected modules with the strongest relevance to fibroblasts as fibroblast-related genes for subsequent analyses.

### 4.6 LASSO–COX regression analysis

The module genes obtained from WGCNA were intersected with 110 DEGs, and 14 fibroblast-related DEGs were identified. LASSO–COX regression analysis was performed based on the fibroblast-related genes using the R "glmnet" package. The parameters in "glmnet" function were set to "family = cox," and "maxit = 1000." We then constructed a fibroblast-related prognostic model for the best genes obtained from the regression analysis, as follows:

Riskcore=ExpressionofGene1×CorrespondingCoefficientGene1+⋯+ExpressionofGenen×CorrespondingCoefficientGenen.

### 4.7 Construction and evaluation of prognostic model

The model genes were mapped using the R "RCircos" package [63] to demonstrate their chromosomal localization. The prognostic status of the model at one to three years was determined using the nomograms. Differences in prognostic outcomes between the different risk score groups are represented as survival plots. The predictive abilities of the model at one, two, and three years are presented using timeROC plots. The weight in "timeROC" function was set to "Marginal". The number of simulations was set to 2000. The "confint" function was used to calculate the 95 % CI of time-dependent AUC estimators. Differences in prognostic evaluation between the prognostic model and other clinical factors were evaluated using multifactorial Cox analysis. The Leuven, Belgium (64 patients) cohort, which contained completely independent data, was used as a validation set to evaluate the prognostic model.

### 4.8 Characterization of high-risk-score and low-risk-score groups

We calculated the infiltration abundance of 22 immune cells in the IPF samples using the CIBERSORT method [64]. We plotted the immune infiltration in the high- and low-risk groups and compared the differences between the two groups. Subsequently, we characterized biological function differences between the different risk score groups using the "GSVA" package [65] via the GO method from the Molecular Signatures Database website (https://www.gsea-msigdb.org/gsea/msigdb) [66]. The KEGG method was used to demonstrate enriched pathway differences between the different risk-score groups.

### 4.9 Statistical methods

Data were analyzed using R version 4.1.2. The Wilcoxon test was used to compare variables. We performed chi-square analysis using Bartlett's test, and Kaplan–Meier (KM) analysis was used for survival curve analysis, with KM curves evaluated using the log-rank test. LASSO–COX analysis was used to construct a regression model. Statistical significance was set at P < 0.05. Pearson's and Spearman's tests were used to calculate the correlation coefficients, with correlation coefficients greater than zero considered positive; the closer the absolute value was to 1, the stronger the correlation.

## Ethical statement

Review or approval by an ethics committee was not needed for this study because our manuscript does not include clinical and animal experiments.

## Funding

Foundation for Innovative Research Groups of the National Natural Science Foundation of China (Grant Nos. 81874442).

## Data availability

The GSE135893 scRNA-seq and GSE70866 bulk RNA-seq datasets analyzed in this study were obtained from the GEO website (https://www.ncbi.nlm.nih.gov/geo/). The code and data used in our manuscript are available from the GitHub website (https://github.com/JiaRui12345/CODE-DATA).

## CRediT authorship contribution statement

**Jiarui Zhao:** Writing – original draft, Visualization, Supervision, Software, Methodology, Investigation, Data curation, Conceptualization. **Chuanqing Jing:** Writing – original draft, Visualization, Validation, Software, Data curation. **Rui Fan:** Writing – original draft, Visualization, Validation, Software. **Wei Zhang:** Writing – review & editing, Validation, Resources, Project administration, Investigation, Funding acquisition, Conceptualization.

## Declaration of competing interest

The authors have no conflict of interest.

## Figures and Tables

**figs1:**
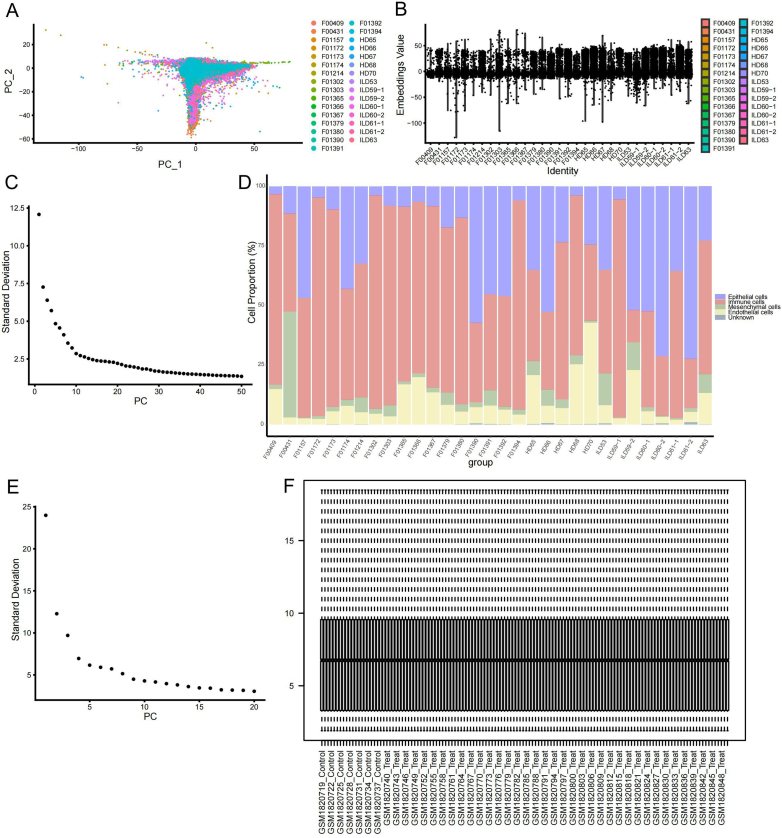


**figs2:**