# Supplementary material for: Prognostic model of fibroblasts in idiopathic pulmonary fibrosis by combined bulk and single-cell RNA-sequencing
Source: Heliyon. 2024 Jul 11;10(14):e34519. doi: 10.1016/j.heliyon.2024.e34519 (PMC11305307; doi:10.1016/j.heliyon.2024.e34519)
Supplement: Multimedia component 8 [file mmc8.docx]

Supplementary Material

Prognostic model of fibroblasts in idiopathic pulmonary fibrosis by combined bulk and single-cell RNA-sequencing

Jiarui Zhao^1^, Chuanqing Jing^1^, Rui Fan^1^, Wei Zhang^1*^

1College of First Clinical Medicine, Shandong University of Traditional Chinese Medicine, Jinan, Shandong, China

*** Correspondence:**Wei Zhang
huxizhijia@126.com

# Supplementary Figures and Tables

## Supplementary Figures


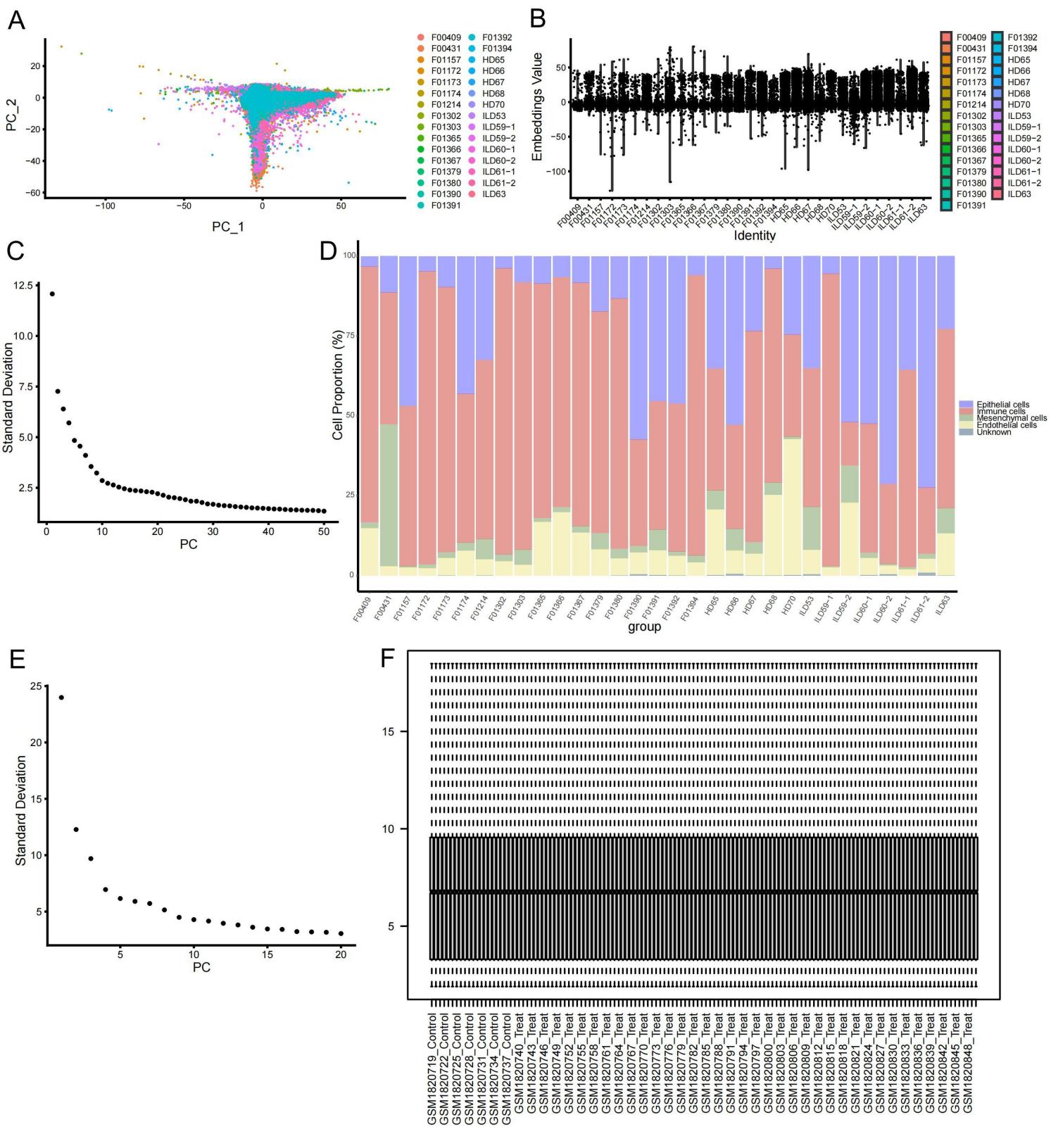


**Supplementary Figure 1.** (A-B) Quality control and preprocessing of scRNA-seq. (C) The PCs were relatively smooth when reduced to 50, a value of 50 was chosen for cell clustering. (D) The distribution of different cell subtypes in the different samples. (E) The PCs were relatively smooth when descending to 20, a value of 20 was chosen for fibroblasts clustering. (F) Quality control of raw bulk RNA-seq data. PC, principal components; RNA-seq, RNA-sequencing; scRNA-seq, single-cell RNA-sequencing.


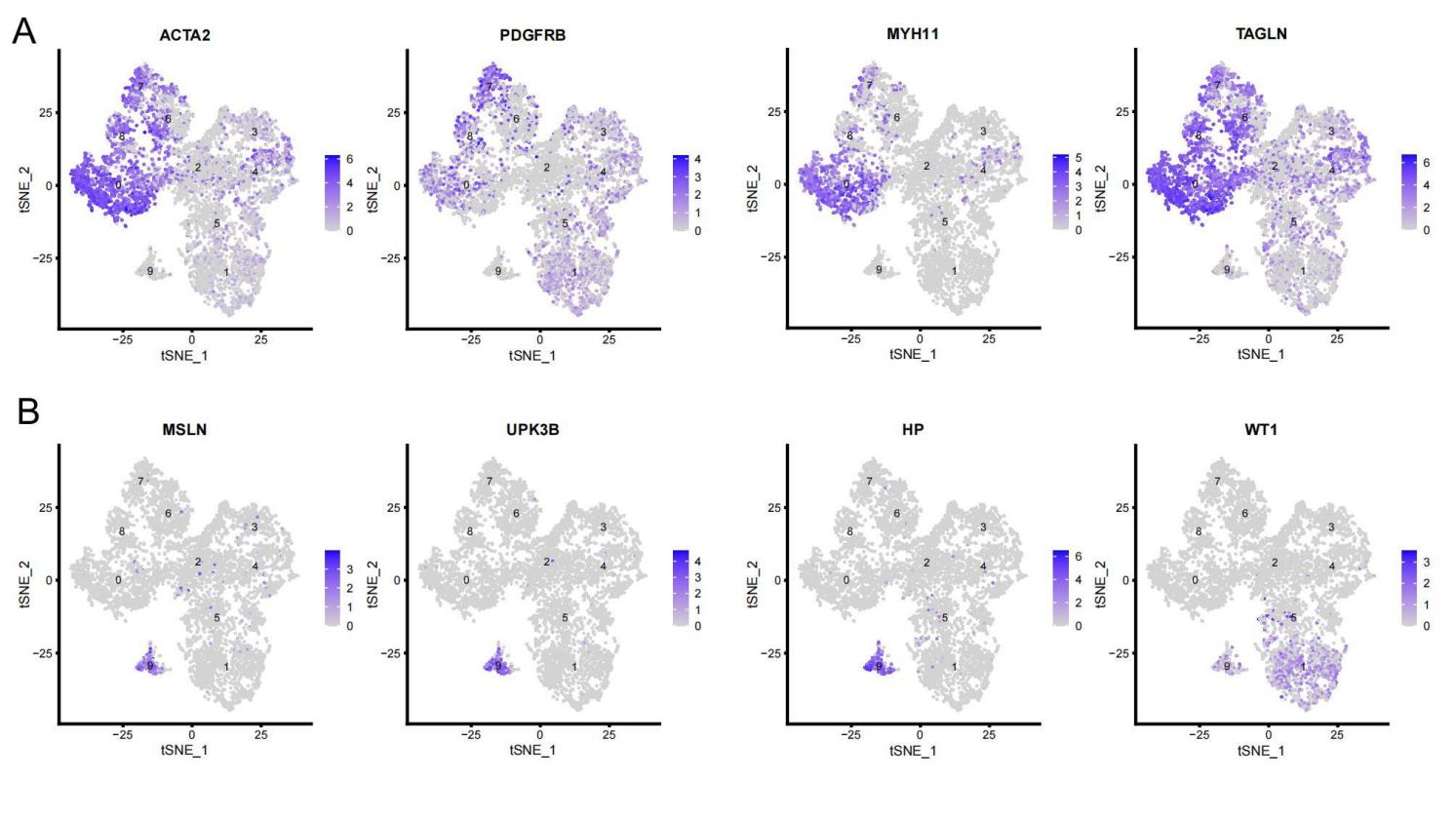


**Supplementary Figure 2.** (A) The marker genes of the smooth muscle cells. (B) The marker genes of the mesothelial cells.

## Supplementary Tables

**Supplementary Table 1.** The marker genes of fibroblasts.

**Supplementary Table 2.** The results of our trajectory from F3+ Fibroblasts to ROBO2+ Fibroblasts.

**Supplementary Table 3.** The results of the differential analysis .

**Supplementary** **Table 4.** The genes of the magenta module.

**Supplementary Table 5.** The 14 fibroblast-related genes .

**Supplementary Table 6.** The clinical factors of the training set.

**Supplementary Table 7.** The clinical factors of the validation set.
